# State Regulation of the Practice of Medicine*Extracts from a paper read before the Buffalo Medical Association, Sept., 1880.

**Published:** 1880-10

**Authors:** William D. Granger


BUFFALO

MEDICAL AND SURGICAL JOURNAL.

VOL. XX.—OCTOBER, 1880.—NO. 3.

### Original Communications.

### STATE REGULATION OF THE PRACTICE OF MEDICINE.*

BY WILLIAM D. GRANGER, M. D.

WITHOUT a law defining what it is to be a physician, any person, whatever his qualifications, or even if he be without any, who styles himself a physician, is one "to all legal intents and purposes." He can exercise the same powers, enjoy the same rights and privileges, and is subject to the same restrictions, whether he has graduated at a school, chartered by the state in which he lives, or whether he never opened a medical book, until he announced himself a "doctor." To-day, in any State without a law regulating the practice of medicine, irregular practitioners of all kinds are to be found, and in our larger cities worse than mere ignorance flourishes. Here congregate the imposters, charlatans, abortionists, and abettors to crime, even to the grade of murder, who live under the protection of the law, and are as legally doctors as any in the land. So great has this evil become that laws regulating this matter have been passed in most, if not all

* Extracts from a paper read before the Buffalo Medical Association, Sept., 1880.

the countries of Europe, the British Provinces, and in the States of New Hampshire, Vermont, New York, North Carolina, Illinois, California and Texas, and perhaps some others. In most foreign countries it is the law to have a body appointed to take cognizance of the preliminary education, and make rules for the matriculation of the student, also to regulate the course of study to be pursued, and hold an examination independent of that of the schools, previous to his entering practice. Nothing of the kind has been attempted in this country. The most required here is that a man who desires to practice medicine, shall prove a good character, pass an examination before a state board to show he is fitted to practice, or present a diploma for approval to the proper authorities, or do both. * * * *

The power to enact a medical license law is one of the so-called police powers of the state, which, according to a high authority, embraces "not only the power to preserve public order, but also to establish for the intercourse of citizen with citizen, those rules for good manners and good neighborhood which are calculated to prevent a conflict of rights, and ensure to all the uninterrupted enjoyment of his own, so far as is reasonably consistent with a like enjoyment by others." It must be confessed that the passage of a medical license law does appear to interfere with the rights of individuals. Therefore it falls upon those desiring such a law, to show to the legislature that is asked to pass such a law, or to the court that is asked to sustain such a law, the necessity for the law. It must appear to them that such a law, interfering as it certainly does with the private rights of some persons, is for the benefit of the people. Should it appear to be enacted for the benefit of any class, as for instance the regular medical profession, it would surely be declared illegal. Recognizing this principle, the petitioners for a license law in Massachusetts, endeavored to prove to the legislature of that state last winter, the public necessity for the law they asked for; but they failed to convince the legislators, or remove from their minds that too many private rights would be broken to warrant its passage. In this interesting controversy the petitioners were not medical men, but were entirely from the laity. The movement was started by a branch of the American Social Science Association. They demanded a law to protect the community against the outrageous boldness of the unprincipled quacks of that state, where they exist in large numbers, especially in Boston. This petition was signed by men of the highest standing in the community, and the petitioners brought before the legislative committee the testimony of physicians, boards of health, prosecuting officers, the officers of private and public charitable institutions, death certificates of ignorant or vicious physicians, coroners' cases, and records of convictions in the court, newspaper advertisements, and from other sources attempted to show the necessity for the law, and yet for reasons which will afterwards be given, but largely because of counter petitions, and complaints that the law was intolerant and exclusive, and for the benefit of a few, and that the rights of many would be interfered with, the law was almost unanimously defeated.

An examination of the medical laws of the different states shows that the tendency of legislation is, first, to legislate who are physicians, and to establish rules by which they may be entitled to practice; second, to define what it is to practice medicine; third, to fix penalties for the punishment of those who practice without authority. Under the first head there is generally provided a licensing board, before which all persons desiring to practice shall appear. In some states an approved diploma is necessary. In others, as in Texas, which has the latest and most stringent law, one is not necessary, but all who desire to practice must first pass an examination before the licensing body. In some states each medical society appoints an examining board. In others, a mixed board is appointed from the different societies, who examine on all questions except therapeutics. * * * [Reference was then made to the practical difficulties encountered in attempting to legislate and define who are physicians, and also accurately to define just what it is to practice medicine. Cases were cited, and laws referred to, to show the difficulties involved, and the following conclusion arrived at.] It would appear then, however carefully the law may be worded, it will come upon judges to decide what it is, and to limit the meaning of the term to practice medicine, in accordance with the statute law of the state, and for the jury to decide of the person on trial is guilty in a particular and specified act of breaking the law. * * *

The most interesting phases of this subject in its present state, are, the law lately passed in Illinois, and which has been productive of so much good in that state, and the effort to have a law passed in Massachusetts last winter. * * *

Brushing away detail, the Illinois law, passed in 1877, enacts "that every person practicing medicine in any of its departments shall possess the qualifications required by the act." There are three classes of persons allowed to practice by this law; those holding approved diplomas, those not holding diplomas, and all those who have practiced ten years in the state.

The state board of health is made the licensing body. Persons holding diplomas must present them to the board for verification of their genuineness, and prove under oath they are the person named in the diploma. They then receive a certificate, which with specified personal items must be recorded for the purpose of identification with the County Clerk. Persons *not* holding diplomas must pass an examination before the board, which in the language of the act "shall be of an elementary and practical character, but sufficiently strict to test the qualifications of the candidate as a practitioner." If they receive a certificate, they must register, as must also practitioners of ten years' standing, and *all* others who practice, even if they are not authorized to do so. The law attempts to define practicing medicine as "publicly professing to be a physician, prescribing for the sick, or appending the letters 'M. D.' to one's name."

Midwives come under the clause "practicing medicine in *any* of its departments," and therefore are subject to the same rules in practicing as physicians, and like them, are obliged to license and register. * * *

From the summary of the report of the board, just published, it appears that in 1880 there are one thousand less practitioners of all kinds in the state than in 1877. That there are one thousand seven hundred and fifty less unqualified practitioners. Also that five hundred and fifty persons, who in 1877 were unqualified to practice, have since, by study in the schools qualified themselves to practice in consequence of this law. The diplomas of two hundred and ten schools have been recognized, and four hundred fraudulent diplomas from Philadelphia and other cities refused. Certificates have been issued to five hundred and fifty midwives, and three hundred, unqualified, have ceased to practice.

The great value of this law is, that all physicians, whether licensed or not are obliged to register, and that the power to license is placed in the one body, and that this body, the board of health, has exerted itself to enforce the law. The weak points of the law are, that persons may practice without a diploma, and that persons with one are allowed to practice without being obliged to be examined to prove their fitness to practice. For the true principles of state medical license are, that all candidates for practice should be examined by a state licensing board that is entirely independent of all the medical colleges, in the state, and he also should be obliged to have a diploma from an approved school. The recognition of these two principles are the most distinctive features of the law proposed by the Massachusetts petitioners. * * *

This law provides for the appointment by the Governor of a body of eight physicians and one dentist, chosen from the different chartered medical societies in proportion to their membership. The regular society with twelve hundred members was to have five, the homœopaths two, and the eclectics one on the board.

Before this board all persons desiring to enter the practice of medicine or dentistry must come and pass an examination on all subjects except therapeutics. After passing this examination they must present a diploma for approval, prove three years study and a good character, then, if they are found qualified they shall receive a license to practice. The reason for presenting the diploma after the examination is, that the board may not know to which school of medicine the candidate belongs. The law also provided for the licensing of midwives if found competent to practice. I have spoken only of the principal points in the law. Many of its details are necessary in order to fully understand its method and object. This law seems to be very comprehensive, and, if the board of examiners do their work well, if the prosecuting officers do their duty, and more than all, if public opinion sustains such a law, it would seem, even if all physicians are not made regular, the people at least would be served by educated physicians.

The opposition to the law was determined, and powerful in numbers. The law had considerable newspaper notoriety. It was defended in some newspapers, by the *Boston Medical Journal*, by most physicians, by many public minded citizens, and largely by the clergy. It was opposed by many papers, by some of our oldest and most honored physicians, by all the quacks and their friends, and by many educated and intelligent citizens. Many objected to it that it interfered with the rights of a large number of citizens, that it debarred many from the services of their favorite and trusted physician; that it was class legislation for the benefit not of the public, but for the pockets of a few. Many persons were brought before the legislative committee to testify that after being treated by regular physicians without benefit, or their cases given up as hopeless, they were cured by these very men the law purposed to prohibit practicing. The reasons for the opposition of members of the regular profession are worthy of careful consideration. Objection was made to the board being appointed by the Governor, and so becoming a political office; also, to its being a mixed board. It was said that these men whom we have expelled from our society, and pronounced irregular, are now asked to examine our members, and see if they are fitted to practice, and if so, license them. Quackery, it was said, would be made respectable. That the worst men, being able to pass examinations, would advertise themselves "licensed physicians." That it is impossible by law to make men honest. That the law would be inoperative, that juries would fail to convict, and there would be another unenforced law on the statute books.

The answer to these objections was: that as it was purely a state matter the appointments must and ought to go to the Governor. That a mixed board was necessary because the law was asked for by non-professional men, for their own protection; that the object of the law was not to make regular, but educated physicians; that the weaker societies, who could have easily defeated the bill, showed great confidence in our honor in agreeing to place in the hands of a board, made up of a majority of members bitterly opposed to them, the licensing of their members; that the only bone of contention, therapeutics, was left out of the examination, and therefore, for these reasons, it could not in any objectionable sense be said that these irregular practitioners licensed members of the Massachusetts Society to practice medicine. It was said that license laws were enforced in other states, and it was reasonable to suppose that it could be in this state, while it was further held, that if there were difficulties in the way it only the more became the duty of those interested to make an earnest effort to enforce it, and also to do what was more important, try and create a public sentiment to sustain the law. * * * As I have said the bill did not become a law. * * * It appears then that the subject of state regulation of the practice of medicine is on trial in this country. What is the best law to meet our peculiar wants is yet to be determined. It is doubtful if the public are ready to enforce a law that commends itself to the best judgment of our profession and a large class of our thinking citizens. The subject is beset with many practical difficulties. Some of these I have endeavored to point out to you in this paper.

